# Chicago Pathological Society

**Published:** 1882-08

**Authors:** 


### ARTICLE VI.

### CHICAGO PATHOLOGICAL SOCIETY.

Stated Meeting, July 24, 1882, Dr. H. M. Lyman, President, in the Chair.

### SUNSTROKE.

Dr. H. D. Valin read the following notes on this subject:

In the *American Journal of Medical Sciences*, 1876, p. 265, an autopsy on a case of sunstroke showed that there was blanching of the various organs; the vessels were congested with dark blood; the brain and its meninges were anæmic, with ecchymoses of the blood-vessels. There was an excess of serum in the ventricles (Dr. Arnd).

According to Sir Jos. Fayrer, the various conditions ordinarily called sunstroke are of three kinds: "simple syncope from exhaustion caused by heat; a condition analogous to shock, due to the action of the direct rays of a powerful sun on the brain and cord; and overheating of the whole body from either the sun's rays or from the high temperature produced by them, causing high thermic fever" (*Med. Rec.*, Vol. XX, p. 334). In the last case, grave structural changes are produced in the nerve centers.

The *symptoms* are unconsciousness; a high temperature; stertorous breathing; pulse rapid and full, sometimes slow; convulsions and death. In simple prostration the temperature may be normal, and the skin may be cold and damp.

"Large cities afford nearly all the cases. In New York over 800 cases have occurred in a single week, in 1868" (Hartshorne's Essentials). Insolation is a common result of a sudden and elevated rise in the temperature, and is most common in day laborers, working in the sun, though cases occur indoors also, and even at night.

*Prophylaxis.*—No disease, probably, could be better prevented than this, if it were not for the cupidity which characterizes capitalists in general. The first precaution should be to stop work when the temperature is excessively high.

*Treatment.*—The patient should invariably be placed in the shade, near the spot where he fell, as removal under these circumstances is a dangerous procedure. He should be wrapped in a wet sheet continually sprinkled with cold water, and ice should be applied to the head. Small doses of whisky or brandy should be frequently administered, and some authors highly recommend a few ten grain doses of quinine. Mustard should be applied to the feet, and a purgative enema administered.

Drs. Tomlinson and Murphy, in *The Practitioner*, June, 1880, report marked success from the hypodermic use of apomorphia hydrochlorate, 1–16 gr., in three cases. Others have used atropia in the same manner. But these various treatments seem inferior in value to the use of the wet sheet. Brandy can be given hypodermically if the patient is unable to swallow. Cases where the temperature has been as high as 108 or 110 have sometimes recovered.

*Sequelæ.*—Dr. Fayrer remarked before the International Congress last summer, that complete recovery from sunstroke was rare, and that mania, epilepsy, or other nervous complaints were liable to remain for ever after. Cases of inebriety have arisen from sunstroke, while the latter is especially prone to affect inebriates. The following are enumerated by Beard and Rockwell as sequelæ to insolation: Spinal irritation; insomnia; neurasthenia; neuralgia; epilepsy; nervous dyspepsia; hysteria; paralysis and insanity, and they recommend general faradization, with central galvanization.

Most of the cases which I have seen were of prostration only, and brandy restored the patients. In two or three cases of congestion of the brain from heat, bromide of potassium in large doses gave a good result. It also proved useful in one case in which the temperature was high, and accompanied by delirium. In this case, although the patient recovered, the mind seemed to have been somewhat weakened, afterward.

One case came under my observation in which sunstroke was followed by hepatization of the right lung, deficiency of the mitral valve of the heart, and hæmoptysis. The patient has improved for about a year, and has gained flesh, but is not able to work.

Dr. Flemming had attended a case in the person of a fireman in an engine-room, who fell under the influence of heat, and was severely burned in his fall, but he ultimately recovered from both injuries. He had also seen the case of an officer in the army in India, who recovered from sunstroke, but was afterward insane. He advocated cold sponging, the internal use of ammonia, and believed that nitrite of amyl should be given a fair trial.

Dr. Tebbits said that he had treated a case of sunstroke in a team driver. He applied ice to the chest and head, gave a purgative enema, brandy hypodermically, and digitalis. The patient recovered, but an antecedent gastritis grew worse after the insolation, and did not heal for six months. He had another case in a foundry, where the patient was heat-struck indoors. The same treatment resulted in his perfect recovery. The temperature of these cases had not been very high.

Dr. Meek related the case of a man who was sunstruck every summer for three years, and the affection had been followed every time by a few months of insanity. The patient was out of the asylum just then.

Dr. R. S. Hall remarked that when treating sunstroke, he applied ice to the carotids and to the wrists, in order to cool the blood more readily. He gave digitalis with bromide of potassium internally, and in case the feet were cold he placed them in hot water, or applied mustard to them if they were warm.

Dr. H. M. Lyman had some personal experience of sunstroke in 1878, a very warm summer. He was not in good health at the time, and had been deprived of sleep lately. After walking four or five blocks in the forenoon, he became affected by the heat. His temperature rose to 101; he experienced a great thirst, extreme weakness, a feeling of alarm or apprehension, and a blurring of the senses. After a few hours, he had dyspnœa, some difficulty in breathing, and was confined to his bed for several days. At first, cold Vichy water was taken for a drink, and small doses of whisky. When convalescent, sailing on the lake proved most beneficial.

The doctor remarked that most cases of insolation occurred in people who had not felt well for some time, or whose constitutions were below par. Sunstroke would not be likely to occur as long as the perspiration escaped freely from the body, as this was a sort of regulating apparatus of the head of the person. He remarked that, as Mr. Dupuy had lately used ether in hypodermic injections in the collapse of cholera, the same might be tried in cases of depression from heat.

Dr. Lyman said that as vomiting and purging sometimes followed sunstroke, he thought this was especially the case in children, and that many cases of death were ascribed to cholera infantum, whereas they resulted from overheating. Claret wine in cold water was a good drink in those cases, and the white of an egg could be added to it. Removal to the country in such cases was a useful means of treatment.

Dr. Meek said that a few years ago, when sunstroke had prevailed in St. Louis, cigar-makers and others who worked in tobacco had been most readily affected, on the same principle, perhaps, that inebriates were so readily affected by the same causes.

Dr. Jos. Haven presented the heart of a male, aged seventeen, which was considerably hypertrophied. The mitral valve was insufficient on one side, and the disease had run its course, it seemed, in two weeks, ending with general œdema. The heart weighed twenty-one ounces. Deceased had never been strong.

